# Are there innate differences in 3D ejection fraction between the sexes detectable by CMR? A CMR study in ~4000 patients

**DOI:** 10.1186/1532-429X-11-S1-P244

**Published:** 2009-01-28

**Authors:** Robert WW Biederman, Diane A Vido, Geetha Rayarao, June A Yamrozik, Ronald B Williams, Saundra B Grant, Mark Doyle

**Affiliations:** Allegheny General Hospital, The Gerald McGinnis Cardiovascular Institute, Pittsburgh, PA USA

**Keywords:** Ejection Fraction, Normal Threshold, Lower Ejection Fraction, Interobserver Reproducibility, Normal Ejection Fraction

## Introduction

It has long been assumed that the standard mechanical contraction parameter ejection fraction (EF) is the same in male and female hearts. Until recently, the fidelity and reproducibility of imaging techniques guaranteed virtual superimposition of EF, nullifying any gender differences were they to be present. The influence of gender on normal thresholds for EF by CV MRI is unknown.

## Hypothesis

Using high resolution 3D CV MRI, we tested the hypothesis that females will have lower EF compared to males.

## Methods

A database composed of consecutive patients who underwent CV MRI scanning (GE, EXCITE HD 1.5 T, Milwaukee, WI) between Aug 2003 and May 2008 was interrogated to yield all patients with EF >55% and no valvular heart disease >1+, no evidence of CAD, HTN, or cardiomyopathy. EF was determined primarily by standard FIESTA 3D methodology or 2D when available. Patients were stratified only by gender and age. We performed two-sample *t* tests to analyze the data and considered differences significant at p < 0.05.

## Results

A total of 3,962 consecutive patients were evaluated, from which 1203 (30%) with normal EF were identified. Mean age was 51.2 ± 18.9 years, 49.4% were males and 50.6% were females. The mean EF for males was 63.6 ± 3.9; range: 56 – 70%, while for females it was 64.3 ± 3.6; range: 56 – 70%, p < 0.001. Under the assumption that EF>55% may not be appropriate for normal thresholds, EF>60% and 65% were also stratified but did not yield significant gender differences. Similarly, stratification by age categories (decades) did not reveal a significant difference. A subset of 150 pts with clinical CV disease, representing the entire range of EF (5%–81%), underwent intra and interobserver reproducibility for LVEF and was 0.13 and 0.85%, respectively.

## Conclusion

Contrary to conventional doctrine, LV ejection performance (EF) as measured clinically, using highly reproducible and accurate 3D CV MRI, is lower for males than females as determined in the largest CV MRI database (3962 patients) to our knowledge to date examining this subject. Thus, beyond establishing normal ranges for LVEF, these observations have far reaching clinical implications in defining thresholds of normality, as well as belying potential, albeit subtle, intrinsic differences in contractile mechanisms. These results show that normal thresholds for EF using CV MRI are influenced by gender.

